# Supplementary material for: Embodied Learning Through Immersive Virtual Reality: Theoretical Perspectives for Art and Design Education
Source: Behav Sci (Basel). 2025 Jul 7;15(7):917. doi: 10.3390/bs15070917 (PMC12292545; doi:10.3390/bs15070917)
Supplement: Supplementary file 1 [file behavsci-15-00917-s001.zip › behavsci-3686821-supplementary.pdf]

Supplementary Materials

Table S1: Comparative Analysis of Embodied IVR Case Studies in Design Education

Study	IVR Activity	Embodiment Level	Primary Interaction Type	Key Findings	Cognitive Benefits	Limitations/ Challenges	Design Education Applications
Chang et al. (2017)	TASC spatial puzzles	3rd degree	Movement tracking, tangible object manipulation, Spatial transformation	Tangible blocks reduced cognitive load by 31%, 78% of participants used physical gestures, Body used for direct measurement	Enhanced spatial reasoning, Reduced cognitive load, Increased retention of volumetric strategies	Requires physical setup, Limited to spatial reasoning tasks, Complex technical requirements	3D modeling instruction, Architectural model design, Form development training
Mills et al. (2022)	Tilt Brush painting	4th degree	Full-body movement, Haptic interaction, Sensory orchestration	76% of students physically circled around creations, Gestural movements emerged to express emotions, Students reported tool embodiment	Enhanced creative expression, Improved perspective taking Better proprioceptive drawing	Immateriality paradox, Initial haptic disorientation, Lack of tactile resistance	Exploratory art education, Spatial visualization Motion sketching
Wang et al, (2019)	'Breaking walls' metaphor	3rd degree	Navigation, Metaphorical interaction with environment	Higher fluidity, originality, flexibility scores (AUT), Deactivation of inhibitory control, Decreased constraints	Enhanced divergent thinking, Reduced cognitive load, Improved cognitive flexibility	Limited to spatial metaphor, May not transfer to creation processes, May not enable flexible creation	Problem-framing techniques, Creative constraints,Divergent thinking training
Leung et al, (2012)	'Thinking outside the box'	3rd degree	Physical positioning, Metaphorical environment	Physically standing outside box increased creative problem-solving, Improved originality, Increased divergent thinking	Embodied conceptual expansion, Enhanced creative problem-solving, Reduced cognitive fixedness	Limited to specific metaphors, Effects may diminish with repeated exposure, Uncertain long-term impacts	Design thinking training, Ideation exercises, Metaphor-based education
Slepian & Ambody (2012)	Movement tracking	2nd degree	Directed tracing, Fine motor control	Tracing fluid curves increased creative association,Improved originality compared to angular movements	Embodied creative output, Improved divergent thinking, Enhanced ideation fluency	Modest effect size, Tablet constraints on IVR, Low efficacy for convergent thinking	Graphic education, Gesture exploration, Virtual gesture tools

Detailed comparison of key case studies examining embodied learning in IVR-based design education, showing embodiment level, interaction types, key findings, cognitive benefits, limitations/challenges, and educational applications.
